# Gender-Pay Equity in Academic Neurosurgery at United States Public Universities

**DOI:** 10.7759/cureus.8655

**Published:** 2020-06-16

**Authors:** Kathryn N Kearns, Ching-Jen Chen, John A Jane, Yashar Kalani, Mark E Shaffrey, Min S Park

**Affiliations:** 1 Neurosurgery, University of Virginia School of Medicine, Charlottesville, USA; 2 Neurosurgery, University of Virginia, Charlottesville, USA; 3 Neurosurgery, Barrow Neurological Institute, Charlottesville, USA

**Keywords:** academic neurosurgery, compensation, gender, equity, public

## Abstract

Background

Compensation has historically been unequal for men versus women in medical fields, particularly in surgical subspecialties.

Objective

We analyzed associations between gender and compensation and identified factors associated with compensation among male and female academic neurosurgeons in the United States (US) public institutions.

Methods

This is a cross-sectional study of available data for the 2016-2017 fiscal years associated with male and female neurosurgical faculty from public, academic institutions within the US. The data used for analysis included total annual salary, which consisted of the base salary and additional compensation. Other gleaned data included faculty demographics, training, and academic appointments. The male and female neurosurgeons' data were separated into two respective gender groups and then were compared. Predictors of compensation were identified using univariable and non-imputed and multiply-imputed multivariable statistical models.

Results

The cohort was comprised of 460 neurosurgery faculty members (female n=34; male n=426). Total annual salaries were comparable between the genders. Females were more likely to be younger (p=0.001), to have completed neurosurgery training recently (p=0.003), to have had fellowship training (p=0.011), and to have lower h­-indices (p=0.003) compared to males. Males and females differed in academic ranks (p=0.035) and neurosurgical subspecialties (p=0.038). Midwest (a$\beta$)=-US$337,516.7, p=0.002), South (a$\beta$=-US$302,500.5, p=0.003), and West (a$\beta$=-US$276,848.8, p=0.005) practices were independent predictors of lower annual compensation. Chair position (a$\beta$=US$174,180.3, p=0.019) and associate professorship (a$\beta$=US$126,633.4, p=0.037) were independent predictors of higher annual compensation. Gender was not a significant predictor of total annual compensation.

Conclusions

Total salaries were not different between male and female neurosurgeons in public, academic institutions in the US. Gender was not a significant predictor of total annual compensation. This study is applicable to public institutions in states with Freedom of Information Act reporting requirements.

## Introduction

Female representation in the field of medicine has grown substantially over the past few decades with women now representing 50% of medical school matriculants - a stark increase from the less than 10% reported previously [1,2]. While this trend is undoubtedly encouraging, female representation remains poor in the historically male-dominated surgical specialties, which include cardiothoracic surgery, orthopedic surgery, and neurological surgery [3,4]. Even in the larger category of science, technology, engineering, and mathematics (STEM) professions, women comprise approximately 48% of the workforce [5]. Studies have observed lower rates of female representation in professions with demanding schedules and competitive environments, such as those commonly experienced in STEM fields [1,4-7]. Lack of female mentors and role models has also been reported by women as deterrents in entering these professions [1,2,4,7,8]. Potential gender-related salary differences in these fields may act as additional disincentives for women [7]. We sought to investigate any potential association between gender and annual compensation and to identify other factors associated with annual compensation among male and female neurosurgeons from public institutions across the United States (US).

## Materials and methods

Study cohort selection

This is a cross-sectional study of publicly available data for the 2016 and 2017 fiscal years from the American Association of Neurological Surgeons (AANS) Neurosurgical Residency Training Program Directory (https://www.aans.org/Trainees/Residency-Directory). The following inclusion criteria were devised: (1) neurological surgery faculties from public, academic institutions within the US; and (2) institutions with publicly-accessible data from state-run databases in accordance with the Freedom of Information Act [9].

The AANS Neurosurgical Residency Training Program Directory described above was then screened for residency training programs at public institutions in states with Freedom of Information act reporting requirements [10]. Programs were selected for inclusion if they had published salaries to review so as to verify that their data was complete. Additional subject information was gathered based on available data published on individual program websites. Faculty from private or foreign institutions as well as those from fellowship-only programs were excluded from the analysis. 

Data collection

Data for analysis was obtained via Internet search in July 2018. Extracted data included faculty demographics, training, and appointment variables. Faculty demographic variables comprised faculty age, gender, and geographic region of practice, the latter of which was categorized into (1) East (Maryland, New Jersey, and New York); (2) Midwest (Illinois, Iowa, Michigan, Minnesota, Missouri, Nebraska, Ohio, Oklahoma, and Wisconsin); (3) North (Vermont); (4) South (Florida, Georgia, Kentucky, Mississippi, North Carolina, Texas, Virginia, and West Virginia); and (5) West (Arizona, California, New Mexico, Utah, and Washington) as decided by the authors. Training variables consisted of time since training completion (residency or fellowship), any advanced degree(s) (in addition to doctor of medicine [M.D.], i.e. doctor of philosophy [Ph.D.]), and any fellowship training. Faculty appointment variables included academic rank (assistant professor, associate professor, or professor), specialty within neurological surgery, chair position, residency program director position, and h-index (via Scopus) [11]. The total annual compensation (reported in US dollars [US$]), which included basic salary and any additional compensation, was used in the analysis. Faculty with incomplete compensation information were designated as missing. To account for varying compensation sources, in which all programs may not be required to report total compensation, a salary of $211,326 was identified, representing 1/2 standard deviation below the mean, and was used as the threshold for inclusion. Programs with faculty salaries below this threshold were designated as having incomplete data. Due to the public availability of the data, institutional review board (IRB) approval and consent were not necessary.

Statistical analysis

All statistical analyses were performed using Stata, version 14.2 (StataCorp LP, College Station, TX). Differences in demographics, training, academic position, specialty, and compensation were compared between male and female neurosurgeons. Continuous variables were compared using Student’s *t* or Mann-Whitney *U* tests, as appropriate. Categorical variables were compared using Pearson’s χ^2^ or Fisher’s exact tests, as appropriate. Linear regression analyses were performed to identify univariable predictors of annual compensation. Univariable predictors of annual compensation with p<0.10 were then entered as independent variables into a multivariable linear regression model to identify independent predictors of annual compensation (model 1). To avoid list-wise deletions due to missing data in the multivariable linear regression model, multiple imputation by chained equations with m=50 was performed. Imputed values for academic rank (3.9%), h-index (3.9%), years out from training (2%), and annual compensation (58.9%) were generated using conditional regression models with the following auxiliary variables: sex, geographic region, chair position, residency program director position, and advanced degree(s). Parameter estimates from analyzing the imputed datasets were pooled according to Rubin’s rules (model 2) [12]. Statistical significance was defined as p<0.05, and all tests were two-tailed.

## Results

Of 1696 neurosurgeons from the 132 American AANS neurosurgery residency programs, 460 faculty members from 39 institutions were included in the study for analysis. The study cohort compares the faculty demographics, training, and appointment variables between female (n=34) and male (n=426) neurosurgeons (Table 1). Female neurosurgeons were more likely to be younger (mean 45.8 vs. 53.8 years, p=0.001), to have had completed training recently (mean 11 vs. 17.7 years, p=0.003), to have had fellowship training (97.1% vs. 79%, p=0.011), and to have lower h­-indices (mean 11.2 vs. 19.5, p=0.003) compared to male neurosurgeons. Women completed fellowships primarily in pediatric neurosurgery (13/34), spine (7/34), or neuro-oncology (6/34). Distributions of academic rank (p=0.035) and specialty (p=0.038) were also different between female and male neurosurgeons, with males holding more full professorships, and completing more vascular neurosurgery, functional/stereotactic/radiosurgery, and spine fellowships than their female colleagues. Geographic region of practice, advanced degree, chair position, residency program director position, and trauma/critical care specialization were comparable between female and male neurosurgeons. Total annual salaries were also similar between female and male neurosurgeons (mean US$534,400 vs. US$602,300, p=0.430).

**Table 1:** Comparison of baseline characteristics between male and female neurosurgeons. yrs = years; n = number; SD = standard deviation; US = United States

	Total (n=460)	Female (n=34)	Male (n=426)	p-value
Age, yrs (SD)	53.2 (12)	45.8 (8.1)	53.8 (12)	0.001
Geographic region, n (%); Regional gender distribution				0.617
East	39/460 (8.5)	2/34 (5.9); 2/39 (5.1)	37/426 (8.7); 37/39 (94.9)	
Midwest	113/460 (24.6)	10/34 (29.4); 10/113 (8.8)	103/426 (24.2); 103/113 (91.2)	
North	5/460 (1.1)	1/34 (2.9); 1/5 (20.0)	4/426 (0.9); 4/5 (80.0)	
South	153/460 (33.3)	11/34 (32.4); 11/153 (7.2)	142/426 (33.3); 142/153 (92.8)	
West	150/460 (32.6)	10/34 (29.4); 10/150 (6.7)	140/426 (32.9); 140/150 (93.3)	
Years out from training, yrs (SD)	17.2 (12.3)	11 (8)	17.7 (12.5)	0.003
Advanced degree(s), n (%)	90/460 (19.6)	5/34 (14.7)	85/426 (20)	0.458
Academic rank, n (%)				0.035
Assistant professor	158/442 (35.7)	17/32 (53.1)	141/410 (34.4)	
Associate professor	111/442 (25.1)	9/32 (28.1)	102/410 (24.9)	
Professor	173/442 (39.1)	6/32 (18.8)	167/410 (40.7)	
Specialty, n (%)				0.038
Neuro-oncology	66/415 (15.9)	6/34 (17.7)	60/381 (15.7)	
Vascular Neurosurgery	94/415 (22.7)	3/34 (8.8)	91/381 (23.9)	
Pediatric Neurosurgery	76/415 (18.3)	13/34 (38.2)	63/381 (16.5)	
Functional/ Stereotactic /Radiosurgery	61/415 (14.7)	4/34 (11.8)	57/381 (15)	
Spine	109/415 (26.3)	7/34 (20.6)	102/381 (26.8)	
Trauma/Critical Care	9/415 (2.2)	1/34 (2.9)	8/381 (2.1)	
Fellowship training, n (%)	367/457 (80.3)	33/34 (97.1)	334/423 (79)	0.011
Chair position, n (%)	40/460 (8.7)	3/34 (8.8)	37/426 (8.7)	1.000
Residency program director position, n (%)	36/460 (7.8)	1/34 (2.9)	35/426 (8.2)	0.502
h-index (SD)	18.9 (15.4)	11.2 (11.9)	19.5 (15.5)	0.003
Annual salary, ´US$1,000 (SD)	597.3 (308.5)	534.4 (225.3)	602.3 (314.1)	0.430

We also examined for predictors of total annual compensation in univariable and multivariable models (Table 2). In the univariable model, geographic location of practice in the Midwest ($\beta$=-US$387,382 [-581,256.6--193,508.1], p<0.001), South ($\beta$=-US$332,090.2 [-510,586.8--153,593.7], p<0.001), and West ($\beta$=-US$271,852.2 [-444.157.8--96,546.5], p=0.003) were associated with lower total annual compensation compared to practice in the East. Longer time interval since training completion ($\beta$=US$5,393.3/year [1,238.5-9,548.1], p=0.011), chair position ($\beta$=US$239,517.8 [239,517.8], p<0.001), and higher h-indices ($\beta$=US$6,689.9 [3,803.2-9,576.6], p<0.001) were associated with higher total annual compensation. Academic ranks of associate professor ($\beta$=US$135,683.8 [25,767-245,600.5], p=0.016) and professor ($\beta$=US$234,165.7[133,788.7-334,542.7], p<0.001) were also associated with higher total annual compensation compared to assistant professor. Faculty age and gender were not associated with total annual compensation.

**Table 2:** Predictors of neurosurgeon annual salary. CI = confidence interval; Ref = reference category * Values based on pooled parameter estimates from multiply imputed data using chained equations with m=50.

	Univariable			Model 1 (n=178)			Model 2 (n=460)*		
Variables	bcoefficient	95% CI	p-value	bcoefficient	95% CI	p-value	bcoefficient	95% CI	p-value
Age	3,820	-878.4–8,518.4	0.110	––	––	––	––	––	––
Male	67,883.8	-101,302.7–237,070.2	0.430	––	––	––	––	––	––
Geographic region									
East	Ref	Ref	Ref	Ref	Ref	Ref	Ref	Ref	Ref
Midwest	-387,382	-581,256.6–-193,508.1	<0.001	-356,224.1	-549,243.1–-163,205.1	<0.001	-337,516.7	-545,025.5–-130,008	0.002
North	––	––	––	––	––	––	35,951.2	-329,628.2–401,530.6	0.846
South	-332,090.2	-510,586.8–-153,593.7	<0.001	-312,617.8	-492,200.8–-133,034.7	0.001	-302,500.5	-499,106.4–-105,894.6	0.003
West	-271,852.2	-444.157.8–-96,546.5	0.003	-284,049.7	-455,562.9–-112,536.5	0.001	-276,848.8	-467,804.6–85,893	0.005
Years out from training	5,393.3	1,238.5–9,548.1	0.011	-3,812.2	-9,344.4–1,720	0.176	-3,175.2	-8,702.9–2,352.5	0.254
Advanced degree(s)	20,613.6	-91,201.1–132,428.2	0.717	––	––	––	––	––	––
Academic rank									
Assistant professor	Ref	Ref	Ref	Ref	Ref	Ref	Ref	Ref	Ref
Associate professor	135,683.8	25,767–245,600.5	0.016	137,105	18,771.8–255,438.2	0.023	126,633.4	7,547–245,719.8	0.037
Professor	234,165.7	133,788.7–334,542.7	<0.001	145,088	-12,251.9–302,427.9	0.070	135,166.3	-21,041–291,374	0.089
Specialty, n (%)									
Neuro-oncology	Ref	Ref	Ref	––	––	––	––	––	––
Vascular Neurosurgery	-52,049.6	-203,212.8–99,113.6	0.498	––	––	––	––	––	––
Pediatric Neurosurgery	-109,987.2	-255,555.8–35,581.5	0.138	––	––	––	––	––	––
Functional/ Stereotactic /Radiosurgery	-108,904.4	-269,275.5–51,466.7	0.182	––	––	––	––	––	––
Spine	113,172.5	-25,820–252,164.9	0.110	––	––	––	––	––	––
Trauma/Critical Care	84,569.3	-533,026.3–702,164.9	0.787	––	––	––	––	––	––
Fellowship training	30,273.6	-92,793.7–153,340.9	0.628	––	––	––	––	––	––
Chair position	239,517.8	108,233.4–370,802.3	<0.001	169,151.3	32,323.7–305,978.9	0.016	174,180.3	29,794.3–318,566.3	0.019
Residency program director position	94,161.88	-40,912.2–229,235.9	0.171	––	––	––	––	––	––
h-index	6,689.9	3,803.2–9,576.6	<0.001	3,718.7	-431.9–7869.3	0.079	3,885.7	-212.7–7,984.1	0.063

In the non-imputed and imputed multivariable models, geographic location of practice in the Midwest (non-imputed a$\beta$=-US$356,224.1 [-549,243.1--163,205.1], p<0.001; imputed a$\beta$=-US$337,516.7 [-545,025.5--130,008], p=0.002), South (non-imputed a$\beta$=-US$312,617.8 [-492,200.8--133,034.7], p=0.001; imputed a$\beta$=-US$302,500.5 [-499,106.4--105,894.6], p=0.003), and West (non-imputed a$\beta$=-US$284,049.7 [-455,562.9--112,536.5], p=0.001; imputed a$\beta$=-US$276,848.8 [-467,804.6-85,893], p=0.005) remained significant predictors of lower total annual compensation compared to practice in the East. Chair position also remained a significant predictor of higher total annual compensation in the non-imputed (a$\beta$=US$169,151.3 [32,323.7-305,978.9], p=0.016) and imputed (a$\beta$=US$174,180.3 [29,794.3-318,566.3], p=0.019) models. Time interval since training completion and h­-index were no longer significant predictors of higher total annual compensation in the multivariable models. Associate professor rank was associated with higher total annual compensation compared to assistant professor rank in the non-imputed (a$\beta$=US$137,105 [18,771.8-255,438.2], p=0.023) and imputed (a$\beta$=US$126,633.4 [7,547-245,719.8], p=0.037) models. However, professor rank was not associated with higher total annual compensation compared to assistant professor rank.

## Discussion

While women constituted approximately 47% of the entire workforce in the US in 2015, they only accounted for 24% of employees within the STEM fields [13]. In a 2017 study of female representation in the STEM fields, Kahn and Ginther observed an overall salary gap of 17% between male and female Ph.D. holders [14]. Part of this difference can be attributed to the greater participation of men in more lucrative specialties, such as engineering and mathematics, while women with STEM degrees tend to enter the lower-paying fields of physical and life sciences. Despite greater female representation in the physical and life sciences workforce, an 8% general wage gap and a 9% wage gap within upper-level management in these disciplines still persisted between genders [8]. Broyles et al. compared the average earnings between male and female chemists, and observed a total pay gap of 26%, 17% of which was found to be due solely to employer discrimination [5]. This resulted in female chemists earning US$3,070 less annually than men after accounting for potential contributing factors (education, experience, etc) [5]. Interestingly, the wage gap is smaller in the male-dominated field of engineering, with female engineers earning approximately 7% less per hour than their male counterparts [8].

This trend continued into the realm of medicine as well. Within the discipline as a whole, women represented 25% of all practicing physicians and 34% of full-time medical school faculty members [2]. Jena et al. reported gender differences in compensation at all levels of faculty rank among academic physicians, and found that the annual adjusted salaries of female full professors (US$250,971) were comparable to those of male associate professors (US$247,212), while male full professors earned US$33,620 more than female full professors annually [4]. In surgical subspecialties, women earned US$43,728 less per year than men in the same fields [4]. After adjusting for age, experience, specialty, faculty rank, research productivity, and payments by Medicare, they concluded that the annual salaries of women in academic medicine were 8% lower than those of men, resulting in an unexplained annual discrepancy of US$19,879 [4]. Similarly, Weiss et al. found that women were paid US$50,000 less overall than men when comparing unadjusted annual industry payments to male and female physicians. This gap widened to US$70,000 among surgeons [15]. Surgical subspecialties showed the greatest gender differences in absolute adjusted annual compensation, with men earning US$43,728 more than women with the same qualifications [4]. This difference was most pronounced in the historically male dominated subspecialties, such as orthopedic surgery (absolute difference of US$40,953), hematology/oncology (absolute difference of US$37,793), and surgical obstetrics/gynecology (absolute difference of US$36,390) [4].

There are also identifiable publication biases between men and women that may contribute to general compensation disparity [16]. According to a recent study analyzing the gender gap in academic literature, women are less likely to obtain senior authorships, less frequently publish as first authors in prestigious journals such as Nature, Lancet, BMJ, and the New England Journal of Medicine, and are less likely to be invited to submit articles when compared to their male colleagues [17]. These publication biases may contribute to the lack of women achieving full professorships in academic neurosurgery programs.

Reduced rates of compensation for women were often attributed to the idea that women provide less “human capital,” or productive labor, than men [5]. Women were more likely to take maternity leave or work part-time and were less likely to negotiate for themselves [1,7]. Furthermore, men have been found to be more motivated by monetary compensation when compared to women, who are more likely to be motivated by predictable schedules and flexible hours [6]. While these differences mitigated some of the gender pay gap, approximately 17% of the gap across all STEM fields was attributed to employer discrimination or immeasurable factors [5]. Therefore, no identifiable statistically significant gender disparity in compensation for academic neurosurgeons at public universities in the US as found in the present paper is a rather unique finding.

In addition to our reported gender-pay equity, several aspects of the field of neurosurgery appear to be unique when compared to other surgical subspecialties. Despite having one of the longest and most rigorous training periods, neurosurgery residents consistently have lower rates of burnout and higher personal accomplishment scores than residents and fellows of other specialties [18-21]. When compared with residents of 13 other surgical specialties, neurosurgery residents ranked 12/14 in rates of depression, 12/14 in rates of low mental quality of life, 13/14 in rates of work/home conflict, and 8/14 in overall rates of burnout [19]. Further examination of burnout among neurosurgery residents revealed statistically significant lower rates of components of this syndrome including emotional exhaustion, feelings of depersonalization, and burnout when compared to a control group of residents and fellows from other specialties [20]. Interestingly, there was no statistically significant difference between these results when comparing neurosurgery residents by gender, while differences were reported for general surgery residents [20]. The question must then be asked: what is different about neurosurgeons? 

A recent survey- based study examining grit (defined as continued fortitude in the face of hardship) and resilience (ability to recovery from a setback) and their relationship to burnout in neurosurgery residents reported an inverse relationship between self-reported grit/resilience and burnout [18]. When evaluating this conclusion with the lower reported rates of burnout in neurosurgery residents, it can be determined that neurosurgery residents possess, if subjectively, at least a statistically higher level of perseverance and fortitude when compared to other medical specialty physicians. Perhaps this can be explained by the intense rigor of the neurosurgical residency matching and training processes. It is no secret that neurosurgery has one of the most competitive residency application processes and applicants must necessarily be intensely committed to the field. Therefore, both male and female applicants must have a rather basic understanding of the expected workload before committing. It has also been proposed that the high sense of personal accomplishment reported by neurosurgical residents may be due to the increased amount of time spent caring for acutely ill patients that make significant recovery after surgical interventions [20]. While residents of other surgical subspecialties may not see such profound improvement in clinical status as a direct result of their care, neurosurgery residents undoubtedly have a sense of personal influence in their training. Therefore, the gender-pay difference may not solely be a result of the women in the field, but rather of the general type of person drawn to the field: strong, resilient physicians with a deep passion for their work, who recognize that strength in others.

Regardless, a great deal of credit must be given to the pioneering women who first made their place in the field of neurosurgery and have worked to promote further female participation. We must also consider the greater societal movement away from the “traditional family” where women are expected to stay home. More and more, women are motivated, either internally or externally, to pursue professions previously deemed inappropriate, and “career women” are growing in number and following the example of those first few. The number of women pursuing a career in neurosurgery has been steadily growing. In 2016, 17% of applicants that matched into neurosurgery residencies were women, an increase from 14% in 2014 [22]. Additionally, the desirability of an academic practice may be similar between men and women. Renfrow et al. analyzed 379 female neurosurgery residency graduates from 1964 to 2013 and found that 26% elected to have an academic practice, a percentage comparable to the overall percentage of board-certified neurosurgeons that entered academic medicine (33%) [2,22]. Historically, women have not entered academic neurosurgery or neurosurgery in general, but current trends indicate that more women are entering the field. From this female cohort, 46.2% achieved the rank of assistant professor, 36.3% achieved associate professorship, and 17.6% became full professors [2]. Our study revealed similar rates of professorship with 53.1%, 28.1%, and 18.8% of female academic neurosurgeons achieving assistant, associate, and full professorships, respectively. However, the distributions of academic ranks were still different between male and female neurosurgeons (p=0.035), with 34.4% of male neurosurgeons holding assistant professorships, 24.9%, associate professorships, and 40.7%, full professorships.

National rates of female participation in leadership positions are increasing substantially. As of 2016, more women are holding higher positions, such as neurosurgery department chair, vice-chair, and director positions, and women have exhibited increased participation in national organizations such as the AANS and Council of State Neurosurgical Societies (CSNS) [2]. In fact, women constituted 19.3% of the 2016/2017 AANS Board of Directors, which was recently led by the first female AANS president [2]. While a lack of female mentors and role models was often identified as an explanation for lower rates of women in surgical specialties, the increasing number of women in neurosurgical leadership roles may be contributing to the compensation parity reported in this study and may be one factor in the rising rate of female participation in neurosurgery as a whole [1,2,4,7,8]. Additionally, organizations such as Women in Neurosurgery have made it their mission to increase visibility and availability of mentorship for aspiring female medical students and residents. Because most of the identified differences in our study directly stem from temporal experience (age, time since training completion, and h-index) rather than gender, it is not unreasonable to expect that, as more women enter the field of academic neurosurgery and assume leadership positions, even the unadjusted annual salaries will begin to equalize.

This data was presented as an abstract at the AANS Scientific Meeting. (Abstract: Kearns K, Chen CJ, Kalani Y, Shaffrey M, Park M. Gender-Pay Equity in Academic Neurosurgery at United States Public Universities. American Association of Neurological Surgeons (AANS) Annual Scientific Meeting; 2019) https://www.aans.org/Annual-Scientific-Meeting/2019/Online-Program/Eposter?eventid=48888&itemid=PLENARY_III&propid=45035

Limitations

One significant limitation surrounding this study is the scarcity of available data for academic neurosurgeons and their salaries. This study only analyzes data made publicly available by programs in states adopting the Freedom of Information act. The programs in this study were selected for inclusion based on completed annual compensation data published on these platforms. The collected data was dependent upon full disclosure of the reporting institutions and may have been subjected to reporting bias. Given that sufficient compensation data was only available for 460 of the 1696 neurosurgeons at AANS-accredited programs, there is a question of the generalizability of our findings to the neurosurgical community at large. It is possible that, if data were to be obtained from private programs or those programs that currently have incomplete public salary reports, a gender-compensation disparity might be identified, as has been shown in similar surgical subspecialties. It has certainly been shown that neurosurgeons in private practice make significantly more in annual salary than do academic neurosurgeons [23]. However, the proportions of female neurosurgeons who pursue academic versus private practices are comparable to those of male neurosurgeons, suggesting the possibility of similar compensation equity in private realms as well [2]. Additionally, if a pay difference were to be identified, it would not detract from the encouraging trends showing increased female participation in academic neurosurgery, or indeed in the field of neurosurgery as a whole. Future studies in this vein would certainly benefit from more complete access to salary reporting measures used by individual programs. 

Perhaps the greatest limitation of this study was the low representation of women in our database. Women constituted approximately 7% of our total data set, reducing the power of our statistical analysis. The general difficulties with acquiring complete compensation information mentioned above were magnified in the much smaller female cohort and potentially influenced our analysis and conclusion. However, the challenge of missing data was distributed randomly among gender, with an increased predilection for men, given the overwhelming majority of the male cohort.

We assumed that the most recently published information accurately depicted the current state of each program and individual physician characteristics. We acknowledge that such sources often do not contain the most up-to-date information, which may introduce unmeasured biases in our results. There may also be additional salaries paid to faculty that were not reported in the database and, thus, were left unaccounted. Thus, we set a salary threshold for inclusion in our study to mitigate this factor. Each institution likely has different reimbursement strategies for their clinical faculty. Programs may elect to further compensate faculty with additional responsibilities within the department or institution or those with more clinical and/or academic productivity. This information may be unavailable to those outside of each program. 

We included an imputed multivariable model in our analysis to account for missing salary data within the database and to adjust for potential biases due to this missing data. Although multiple imputation minimizes bias and maximizes the use of available data, these fall under the assumption that the data is missing at random. Lastly, the results of this study are limited to clinical neurosurgical faculty in public, academic institutions, which represent a small subset of neurosurgeons within the US. Additionally, some of the programs included in this analysis did not contain any female faculty members. Thus, our results may not be generalizable to the entire neurosurgical community.

## Conclusions

We did not identify a statistically significant difference in annual compensation among female and male academic neurosurgeons in the US practicing at public universities subject to the Freedom of Information Act. Female neurosurgeons were more likely to be younger, to have had completed training recently, to have had fellowship training, and to have lower h­-indices. Additionally, male and female neurosurgeons differed in academic ranks and specialties. Geography, academic rank, and the position of department chair were independent predictors of compensation differences. The results of this study suggest that the previously identified gender-compensation discrepancies in similar fields may not be as significant in academic neurosurgery. 
